# Acute and late-onset optic atrophy due to a novel OPA1 mutation leading to a mitochondrial coupling defect

**Published:** 2009-03-27

**Authors:** Yannick Nochez, Sophie Arsene, Naig Gueguen, Arnaud Chevrollier, Marc Ferré, Virginie Guillet, Valérie Desquiret, Annick Toutain, Dominique Bonneau, Vincent Procaccio, Patrizia Amati-Bonneau, Pierre-Jean Pisella, Pascal Reynier

**Affiliations:** 1 Centre Hospitalier Universitaire de Tours, Service d'Ophtalmologie, Tours, France; 2 CHU d’Angers, Département de Biochimie et Génétique, Angers, France; 3 INSERM, U694, Angers, France; 4 Université d’Angers, Faculté de Médecine, Angers, France; 5 Centre Hospitalier Universitaire de Tours, Service de Génétique, Tours, France; 6 CNRS, UMR6214, Angers, France; 7 INSERM, U771, Angers, France

## Abstract

**Purpose:**

Autosomal dominant optic atrophy (ADOA, OMIM 165500), an inherited optic neuropathy that leads to retinal ganglion cell degeneration and reduced visual acuity during the early decades of life, is mainly associated with mutations in the *OPA1* gene. Here we report a novel ADOA phenotype associated with a new pathogenic *OPA1* gene mutation.

**Methods:**

The patient, a 62-year-old woman, was referred for acute, painless, and severe visual loss in her right eye. Acute visual loss in her left eye occurred a year after initial presentation. MRI confirmed the diagnosis of isolated atrophic bilateral optic neuropathy. We performed DNA sequencing of the entire coding sequence and the exon/intron junctions of the *OPA1* gene, and we searched for the mitochondrial DNA mutations responsible for Leber hereditary optic atrophy by sequencing entirely mitochondrial DNA. Mitochondrial respiratory chain complex activity and mitochondrial morphology were investigated in skin fibroblasts from the patient and controls.

**Results:**

We identified a novel heterozygous missense mutation (c.2794C>T) in exon 27 of the *OPA1* gene, resulting in an amino acid change (p.R932C) in the protein. This mutation, which affects a highly conserved amino acids, has not been previously reported, and was absent in 400 control chromosomes. Mitochondrial DNA sequence analysis did not reveal any mutation associated with Leber hereditary optic neuropathy or any pathogenic mutations. The investigation of skin fibroblasts from the patient revealed a coupling defect of oxidative phosphorylation and a larger proportion of short mitochondria than in controls.

**Conclusions:**

The presence of an OPA1 mutation indicates that this sporadic, late-onset acute case of optic neuropathy is related to ADOA and to a mitochondrial energetic defect. This suggests that the mutational screening of the *OPA1* gene would be justified in atypical cases of optic nerve atrophy with no evident cause.

## Introduction

Autosomal dominant optic atrophy (ADOA, OMIM 165500) is a hereditary disorder characterized by progressive loss of visual acuity in the early decades of life, color vision deficits, optic nerve pallor, and central or centrocecal visual field scotoma [1,2]. ADOA occurs with an estimated prevalence of 1:50,000 in most populations [3], and 1:10,000 in Denmark [4]. This hereditary optic neuropathy leads to phenotypic heterogeneity, even among members of a given family [5]. Histopathological studies indicate that ADOA is caused by the degeneration of retinal ganglion cells followed by the ascending atrophy of the optic nerve [6].

In 2000, two research groups identified *OPA1* gene mutations on chromosome 3q28 as causing ADOA [7,8]. The *OPA1* gene encodes a dynamin-related guanosine triphosphatase (GTPase) and is composed of 31 exons [9]. As of January 2009, 204 *OPA1* pathogenic mutations have been reported, mainly in the GTPase and the C-terminal domains of the protein (eOPA1) [10]. The OPA1 protein is localized to the mitochondrial intermembrane space, where it facilitates fusion between mitochondria [11]. The protein is involved in several mitochondrial functions, such as the maintenance of the integrity of the cristae formed by the mitochondrial inner membrane [12], the regulation of cytochrome c release during apoptosis [13], and the maintenance of mitochondrial DNA [14,15].

Studies of patients carrying pathogenic OPA1 mutations have revealed the great diversity of clinical presentations of ADOA by the description of congenital forms of the disease [16], of forms with spontaneous visual recovery [17], and forms associated with extraocular symptoms such as deafness [18], polyneuropathy [19], chronic progressive ophthalmoplegia [20], myopathy, and encephalopathy [14,15]. An energy impairment has been found to be associated with the disease in vivo [21] as well as in the patient's fibroblasts [22]. In addition to this energy impairment, it has been shown that the mitochondrial network of mutated fibroblasts was frequently fragmented and that some OPA1 mutations were associated to increased susceptibility to apoptosis [23]. Lastly, patients with severe ADOA with extraocular symptom were found to harbor multiple mitochondrial DNA deletions [14,15], reinforcing the strong links existing between mitochondrial structure maintenance and mitochondrial energetic metabolism in neurodegenerative diseases.

The aim of the present report is to show that a clinical phenotype similar to that of late-onset Leber hereditary optic neuropathy may be linked to an OPA1 mutation. This report, together with other recent studies, reveals the unexpected clinical heterogeneity of ADOA caused by OPA1 mutations.

## Methods

### Patient history

A 62-year-old Caucasian woman consulted for blurred vision. On examination, she was found to have a central scotoma in the right eye; visual failure occurred rapidly a few days later. She denied having had any previous ocular symptoms, ocular pain, or systemic symptoms such as headache, muscular pain, or jaw claudication. An ophthalmic examination documented a right optic disc edema without peripapillary hemorrhage. No morphological abnormalities in optic disc cupping or loss of the neuroretinal rim were found in either eye. The visual acuity in the right eye, which had a large central scotoma, fell to 20/400, but remained at 20/20 in the left eye. The intraocular pressure in each eye was 15 mmHg.

The patient had no known vascular risk factors. She showed no signs of hypertension, diabetes, or hyperlipidemia, and did not indulge in alcohol or tobacco. The electrocardiogram was normal, without any signs of dysrythmia. The carotid and the transcranial Doppler scans showed no prominent stenosis or occlusion of the major intracranial large arteries. The general and neurologic examinations were normal. Brain MRI was normal. Laboratory investigations were done to exclude other causes of arteritis, such as syphilis, hepatitis, Lyme disease, or systemic lupus erythromatosis. The erythrocyte sedimentation rate and the C-reactive protein level were not elevated. A temporal artery biopsy, done to check for inflammatory arterial cells, was normal. As the patient was suspected of having giant-cell arteritis and arteritic anterior ischemic optic neuropathy, she was treated with 500 mg I.V. methylprednisolone for three days, while awaiting biopsy results, to prevent vision loss from progressing to the other eye. Visual acuity remained 20/400 in the right eye, with resolution of the disc edema.

Nine months after the loss of vision in the right eye, vision in the left eye became blurred and worsened to 20/250 over the following week. On ophthalmoscopic examination, the left optic disc showed edema without splinter hemorrhages at the disc margin. The swelling of the optic disc gradually subsided and turned pale. The same clinical, biologic, and radiological investigations as before showed no abnormalities. Brain and orbital MRI was normal. Our hypothesis was that the patient had an acute anterior ischemic optic neuropathy, caused either by an inflammatory involvement of the optic nerve or by an ischemic sequela to vasculitis. Since there may have been some overlap between the two conditions, the patient was treated with intravenous methylprednisolone to try to improve her vision. Nevertheless, the visual acuity remained at 20/250 in the left eye, with resolution of the disc edema.

**Figure 1:**
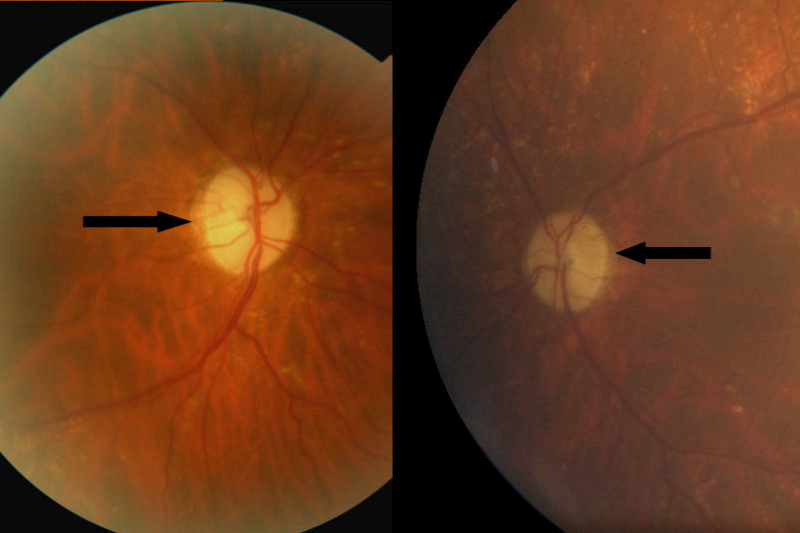
Fundus examination. The first image represents the patient's right eye fundus examination and we observe optic disk atrophy (arrow). The second image represents left eye fundus examination, we also observe optic disk atrophy.

At the last examination six months later, the patient, still amaurotic, was given a poor prognosis for further recovery of the visual defects. Fundus examination revealed severe optic disc atrophy in both eyes (Figure 1). The clinical examination and the MRI confirmed the diagnosis of isolated bilateral optic neuropathy. We searched for a genetic etiology. The patient’s parents were dead; she had no brothers or sisters; but she had a 28-year-old son who had no ophthalmic problems.

### *OPA1* gene analysis

Blood samples were taken from the patient after obtaining informed consent. Genomic DNA was extracted from blood samples using the BioRobot EZ1 and the EZ1 DNA Blood kit (Qiagen, Courtaboeuf, France). Next, 30 primer pairs (Appendix 1) were used to amplify the 30 *OPA1* coding exons, including exon-intron junctions. PCR amplifications of the DNA were conducted under standard protocols. The purified PCR products were sequenced using a Ceq2000 DNA sequencer (CEQ DTCS-Quick Start kit; Beckman Coulter, Fullerton, CA). The *OPA1* mutation is described according to the *OPA1* transcript variant 1 (RefSeq: NM_015560).

### Mitochondrial DNA analysis

To exclude the presence of any rare mitochondrial DNA mutation, we sequenced entirely the mitochondrial genome. The mtDNA was PCR amplified in eight fragments using the protocol 96 °C for 10 min, followed by 30 cycles of 96 °C for 45 s, 58 °C for 30 s, 72 °C for 3 min, and a last extension at 72 °C for 10 min (Appendix 2). PCR products were purified and sequenced as described in the previous section.

### Skin fibroblasts

The research followed the tenets of the Declaration of Helsinki. Fibroblast cells were obtained from skin biopsies taken after obtaining written consent from the patient, as described elsewhere [24]. Fibroblasts, obtained by explants from skin punch biopsy, were maintained in DMEM (Invitrogen, Carlsbad, CA) with 10% bovine calf serum at 37 °C in a humidified atmosphere with 5% CO_2_. All fibroblast cultures were mycoplasma-free, as shown by the DAPI/Hoechst in situ coloration and by PCR (Venor®Gem, BioValley, Marne-la-Vallée, France). All experiments were performed on cells with similar passage numbers, ranging from 5 to 15, so as to avoid artifacts due to senescence, known to occur at passage numbers greater than 30.

### Efficiency of mitochondrial ATP production in permeabilized cells

The rate of mitochondrial ATP synthesis and the ATP/O ratio were determined in cells permeabilized by exposure to digitonin, as described below [24]. Cells were resuspended in the respiratory buffer, which contained 10 mM KH_2_PO_4_, 300 mM mannitol, 10 mM KCl, and 5 mM MgCl_2_, pH 7.4. This buffer was supplemented with 2 mM iodoacetate and 2 mM EDTA so as to prevent glycolytic ATP synthesis and ATP hydrolysis by cellular ATPases. The respiratory rates (O_2_/min/mg of proteins) of 3–5x10^6^ cells were recorded at 37 °C in 2 ml glass chambers using a two-channel, high-resolution Oxygraph respirometer (Oroboros, Innsbruck, Austria). ATP synthesis was started by addition of 5 mM malate, 5 mM pyruvate, and 10 mM succinate, followed by addition of 1.5 mM ADP. Five aliquots were sampled each minute during mitochondrial ATP synthesis, quenched with an equal volume of 1%(W/V) TCA solution and neutralized by adding a 25 mM HEPES, 2 mM EDTA, pH 7.75 buffer. Three aliquots were also sampled after addition of 8 µg/ml oligomycine (an ATP synthase inhibitor) to check for residual nonmitochondrial ATP synthesis. The ATP synthesized in situ was measured using the Enliten ATP assay (Promega, Madison, WI). Luminescence was measured on a multidetection reader for microplates Xenius XML (SAFAS, Monaco, Monaco) using a 10-s integration period. Standardization was performed with known quantities of ATP measured under the same conditions. Coupling efficiency (ATP/oxygen [ATP/O]) was measured by polarography as the number of nanomoles of ATP produced from ADP+Pi per nanomole of oxygen consumed by permeabilized cells. The efficiency of the respiratory chain was tested using malate, pyruvate, and succinate as substrates.

A Beckman DU 640 spectrophotometer (Beckman Coulter, Fullerton, CA) was used to measure the activity of the mitochondrial respiratory chain complexes on cell homogenates at 37 °C in a cell buffer that contained 250 mM saccharose, 20 mM tris[hydroxymethyl]aminomethane, 2 mM EGTA, and 1 mg/ml BSA, pH 7.2. Complex IV (cytochrome c oxidase, COX), complex V (F1-ATPase), and citrate synthase activities were recorded following Rustin et al. [25]. Complex IV activity was measured in a 50 mM KH_2_PO_4_ buffer, using 15 µM reduced cytochrome *c* on 10^5^ cells permeabilized by 2.5 mM β-D-dodecylmaltoside. To measure the activity of complex V, we first disrupted cells by freezing in liquid nitrogen, followed by rapid thawing at 37 °C. The cells were then centrifuged for 2 min at 800× *g*, resuspended in cell buffer (250 µl/10^6^ cells). This was followed by a sonication step (6×5 s with an MSE sonicator). Complex V activity was immediately assayed on this cell lysate (0.5×10^6^ cells) in a Tris/KCl buffer, composed of 50 mM Tris and 10 mM KCl, and containing 2 mM Phosphoenolpyruvate (PEP), 0.5 mM ATP, 5 mM MgCl_2_, 1.5 µM FCCP, 2.5 µg/µl antimycin A, 0.1 U lactate dehydrogenase, and 0.1 U pyruvate kinase (Roche Diagnostics, Meylan, France), 5 mg/ml BSA, pH 8. After incubation for 3 min, the reaction was started by adding 0.1 mM NADH and the rate of disappearance of NADH was monitored at 340 nm. In addition, 10 µM oligomycin was added to determine the background rate (nonspecific for complex V activity). Citrate synthase was assayed by standard procedures, using 0.15 mM 5–5-dithiobis(2-nitrobenzoic acid) (DTNB), 0.5 mM oxaloacetate, 0.3 mM acetyl-CoA, and 0.1% (V/V) Triton X-100. Specific enzymatic activities were expressed in mIU (e.g., nanomoles of cytochrome *c,* NADH or DTNB/min/mg protein, respectively). Enzymatic activities of complexes IV, V and citrate synthase are listed in Table 1.

**Table 1:** Enzymatic activities of complexes IV, V and citrate synthase.

**Cells**	**Complex IV**	**Complex V**	**CS**	**Complex IV/CS**	**Complex V/CS**
controls	256±40	123±31	362±75	0.68±0.12	0.38±0.11
ADOA	185±44	214±52	363±65	0.51±0.08	0.62±0.15
OPA1_R932C	105	200	368	0.29	0.54

The enzymatic activities were measured in three experiments for the patient and each control subject (n=6). Results are expressed as mIU (nmol substrate/min/mg protein), and normalized to citrate synthase. Results are given as mean±standard deviation.

### Mitochondrial fluorescence imaging

Cells cultured in a two-well chamber slide (Labtek, Nunc International, Naperville, IL) were incubated with 100 nM Mitotracker® to label the mitochondrial network (Molecular Probes, Carlsbad, CA) according to manufacturer’s instructions. Z-Stack fluorescent images were acquired with a Leica (DMI6000B; Microsystems GmbH, Wetzlar, Germany) and a Roper CoolSnap HQ2 camera. MetaMorph software (Molecular Devices, Sunnyvale, CA) was used to analyze images. Mitochondrial length and number were determined using integrated morphometric analysis of the regions created around mitochondria after deconvolution (Metamorph®, Molecular Devices), as described above by Cassereau et al. [26]. Around 50 deconvolved images were used to quantify the mitochondrial length. Measurements of mitochondria length distribution were divided into four groups: <1 µm, 1 to 5 µm, 5 to 10 µm, and >10 µm.

### Expression of OPA1 protein

Pellets of 5×10^6^ fibroblasts from the patient and controls were stored at −80 °C until used for western blot analysis. Next, 40 µg protein were solubilized in Laemmli buffer and heated for 5 min at 50 °C. Proteins were separated on an 8% SDS-polyacrylamide gel and electroblotted to a PVDF membrane (Amersham Biosciences, Buckinghamshire, UK). Membranes were saturated overnight at 4 °C with 5% nonfat milk dissolved in TBS-Tween-0.1%, pH 7.4, which contained 137 mM NaCl, 2.7 mM KCl, 23 mM Tris, and 0.1% Tween-20. Membranes were then incubated 2 h at room temperature with monoclonal mouse anti-OPA1 antibody (BD Bioscience PharMingen, Milan, Italy) and mouse monoclonal anti-Hsp60 antibody (Stressgene, Victoria, Canada). Membranes were then washed three times in TBS-Tween-0.1% and incubated with 1:10,000 horseradish peroxidase-conjugated rabbit anti-mouse secondary antibody for 1 h at room temperature. The immunoreactive proteins were visualized with enhanced chemiluminescence (ECL Plus Western Blotting Detection Reagents; Amersham Biosciences). Band intensities were quantified with Quantity One software (Bio-Rad, Hercules, CA).

## Results

**Figure 2:**
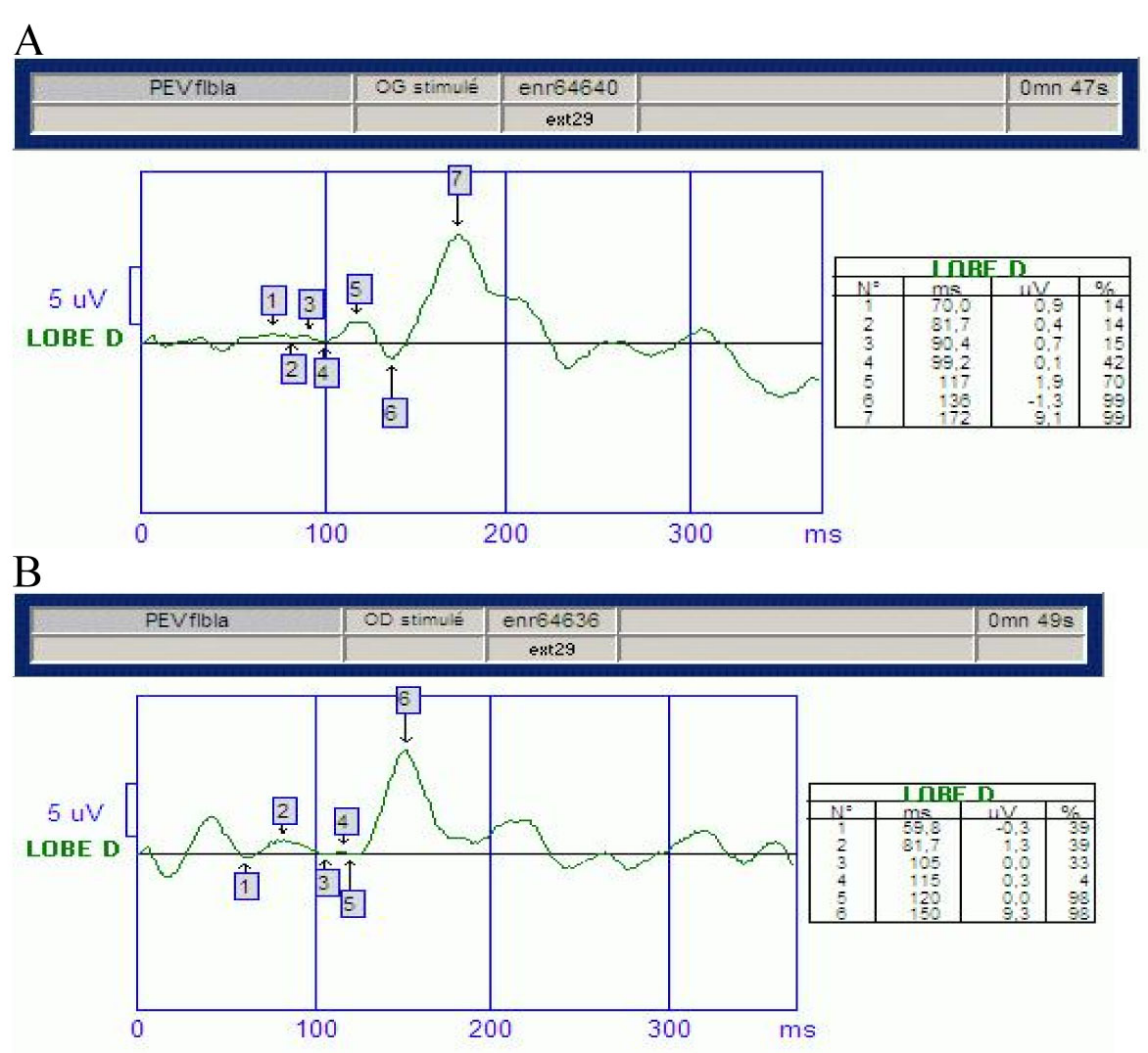
Patient 's visual evoked potentials. We observe low-amplitude potentials and increased latency in visual evoked potentials in left eye (**A**) and in right eye (**B**). It indicates defective conduction of the optic nerves.

**Figure 3:**
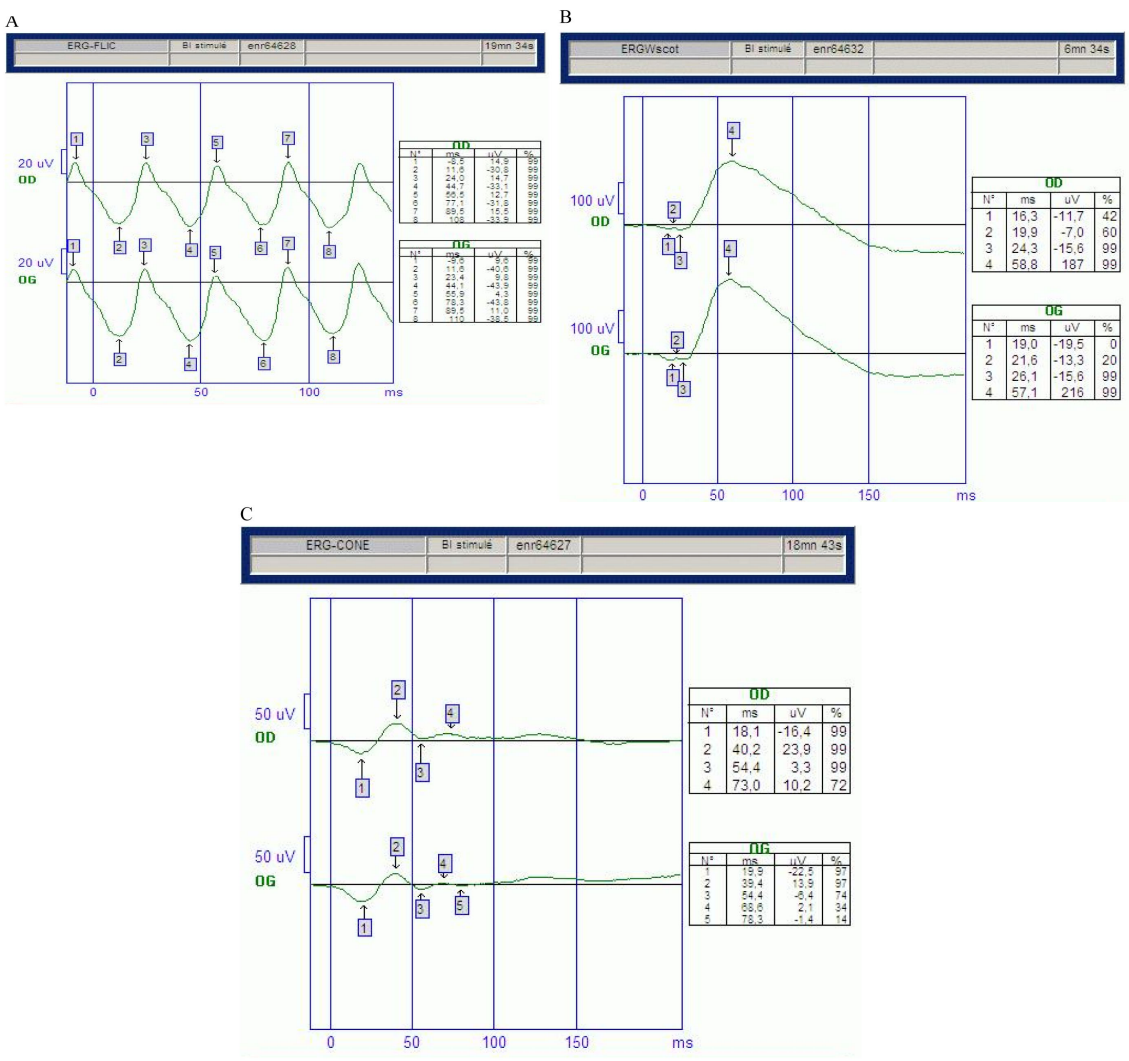
Patient's electroretinograms. We show normal flicker electroretinograms (**A**), scotopic electroretinograms (**B**), and photopic electroretinograms (**C**).

**Figure 4:**
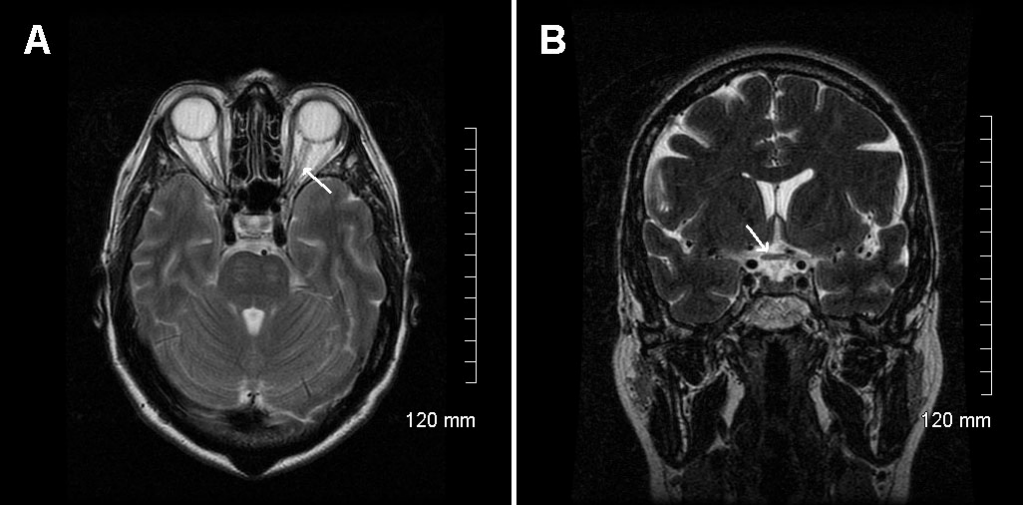
Patient's encephalic MRI. MRI of the optic nerve showed severe bilateral atrophy of the optic nerve from retina to the lateral geniculate nucleus, seen on horizontal MRI section (**A**), including atrophy of the optic chiasma, seen on coronal MRI section (**B**).

The clinical examination and MRI confirmed the diagnosis of isolated bilateral optic neuropathy in our patient. Visual fields showed large centrocecal defects and central visual field loss. Visual evoked potential to a bright flash showed low-amplitude potentials and increased latency in both eyes, indicating defective conduction of the optic nerves (Figure 2). Flash-electroretinograms and flicker electroretinograms were within normal limits (Figure 3). Color-axis confusion lines were not specific because of many mistakes due to low visual acuity. MRI of the optic nerve showed severe bilateral atrophy of the optic nerve from retina to the lateral geniculate nucleus including atrophy of the optic chiasma and the optic tract (Figure 4). On T2-weighted and FLAIR acquisitions, no white matter lesions were found. On orbital high-resolution MRI with fast spin-echo, there was no obvious change in signal intensity in the retrobulbar portion of the right optic nerve.

The analysis of the *OPA1* gene revealed a sporadic heterozygous missense mutation in exon 27: c.2794C>T. This mutation has not been previously described. The substitution, which was not found in 400 control chromosomes, is likely to be causative of disease since it leads to an amino acid change (p.R932C) in a strongly conserved C-terminal domain of the OPA1 protein. This domain is predicted to form a coiled-coil domain, allowing interactions between OPA1 proteins. To rule out the possibility of an mtDNA variant responsible for the disease phenotype, we sequenced the patient’s mtDNA, which revealed an H haplogroup containing 14 nucleotide differences relative to the Cambridge reference sequence [27]. No known or potential pathogenic mtDNA variants were identified. One change at position m.2363A>G in 16S rRNA has never been seen before in mitochondrial DNA databases. So far, the large majority of the putative LHON pathogenic mutations that are associated with LHON are located in complex I subunits. This homoplasmic variant in 16S rRNA appears very unlikely to be a primary mutation responsible for the disease phenotype. The identified variants are listed in Table 2.

**Table 2:** Sequence variations of the patient’s mitochondrial genome.

**Nucleotide position**	**Amino acid substitution**	**Locus**	**Presence in Mitomap database**	**Status**
m.263A>G	-	MT-DLOOP	Yes	Polymorphism
m.310_311insC	-	MT-DLOOP	Yes	Polymorphism
m.750A>G	-	MT-RNR1	Yes	Polymorphism
m.1438A>G	-	MT-RNR1	Yes	Polymorphism
m.2363A>G	-	MT-RNR2	No	Unknown
m.3010G>A	-	MT-RNR2	Yes	Polymorphism
m.4769A>G	p.M100M	MT-ND2	Yes	Synonymous change
m.8860A>G	p.T112A	MT-ATP6	Yes	Polymorphism
m.9966G>A	p.V254I	MT-CO3	Yes	Polymorphism
m.15326A>G	p.T194A	MT-CYB	Yes	Polymorphism
m.15742C>A	p.L332L	MT-CYB	No	Synonymous change
m.16092T>C	-	MT-DLOOP	Yes	Polymorphism
m.16297T>C	-	MT-DLOOP	Yes	Polymorphism
m.16519T>C	-	MT-DLOOP	Yes	Polymorphism

The patient's mtDNA was entirely sequenced. The sequence was compared to the revised Cambridge reference sequence of Human mtDNA. The patient was found to carry 14 nucleotide variations. No known or potential pathogenic mtDNA mutation were identified.

**Figure 5:**
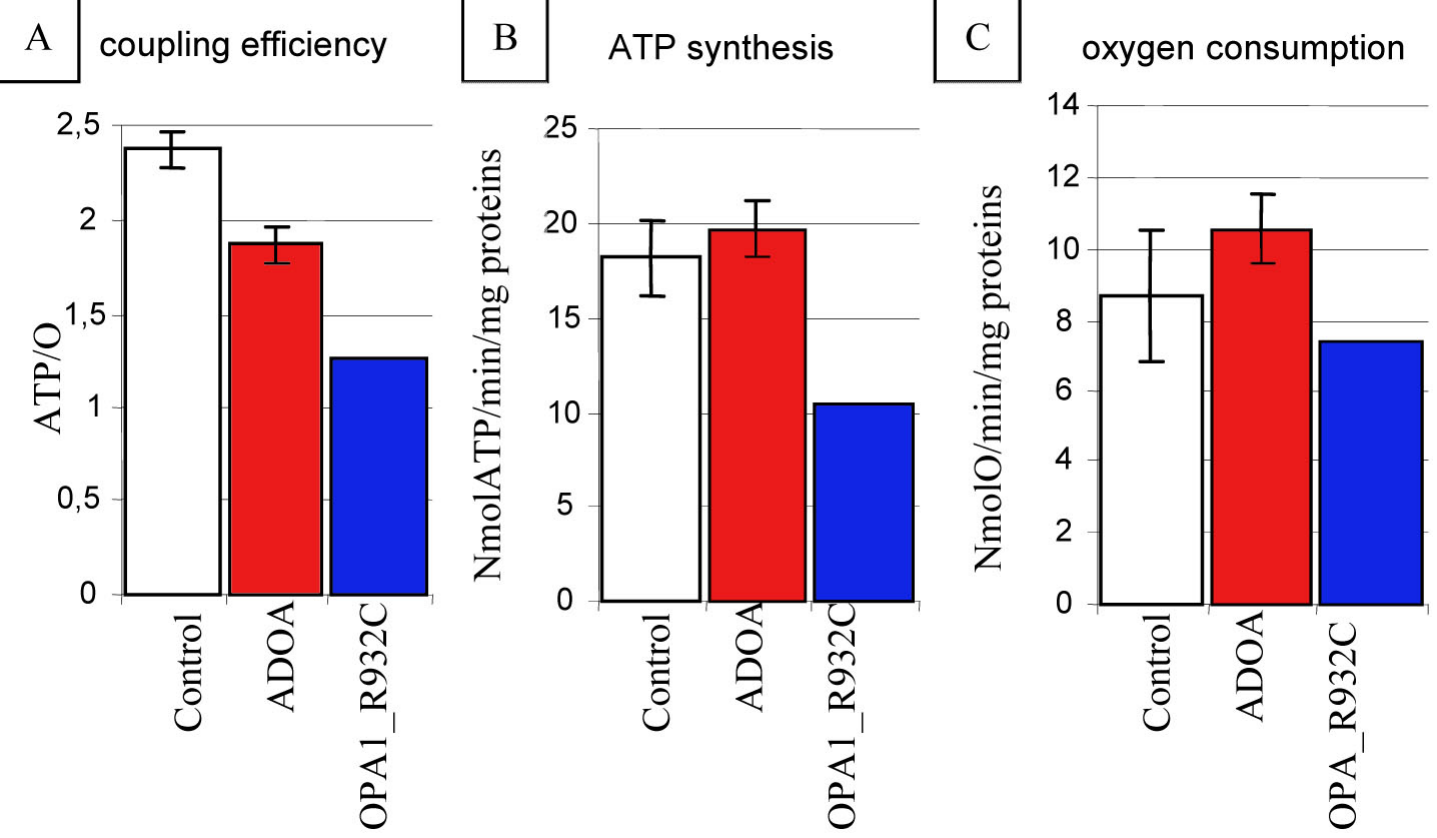
Mitochondrial metabolic investigations in the patient and controls. **A:** Coupling efficiency (ATP/O): Reduced efficiency of ATP synthesis was found in the patient’s fibroblasts in comparison to controls. **B:** Reduced mitochondrial ATP synthesis, measured by luminescence from aliquot samples in a polarographic chamber, was found in the patient’s fibroblasts in comparison to controls. **C:** Maximal coupled respiration rates, i.e., ADP-stimulated oxygen consumption with malate, pyruvate, and succinate, was measured in the same polarographic chamber.

Biochemical analysis of the patient’s fibroblasts revealed mitochondrial energetic defects, thus confirming the pathogenicity of the OPA1 mutation (Table 1). There was a deficiency in the activity of the terminal enzyme of respiratory chain, COX. The COX/citrate synthase ratio in patient’s fibroblasts was 0.29 versus 0.66±0.12 (n=6) in controls (Table 1). The activity of complexes I, II, III, and V in patient’s fibroblasts did not differ from that in controls. Mitochondrial ATP synthesis was greatly decreased in the patient’s fibroblasts compared to controls either using malate, pyruvate, and succinate (maximal coupled respiration state; Figure 5B) or pyruvate plus malate (complex I – related coupled respiration, not shown). This is probably due to the reduced efficiency of ATP synthesis: the ATP/O in the patient’s fibroblasts was 1.26 versus 2.37±0.10 in controls (n=5) using malate, pyruvate, and succinate as substrates (Figure 5A), and 1.01 in the patient's cells versus 2.04±0.15 in controls (n=5) using malate and pyruvate (data not shown)

**Figure 6:**
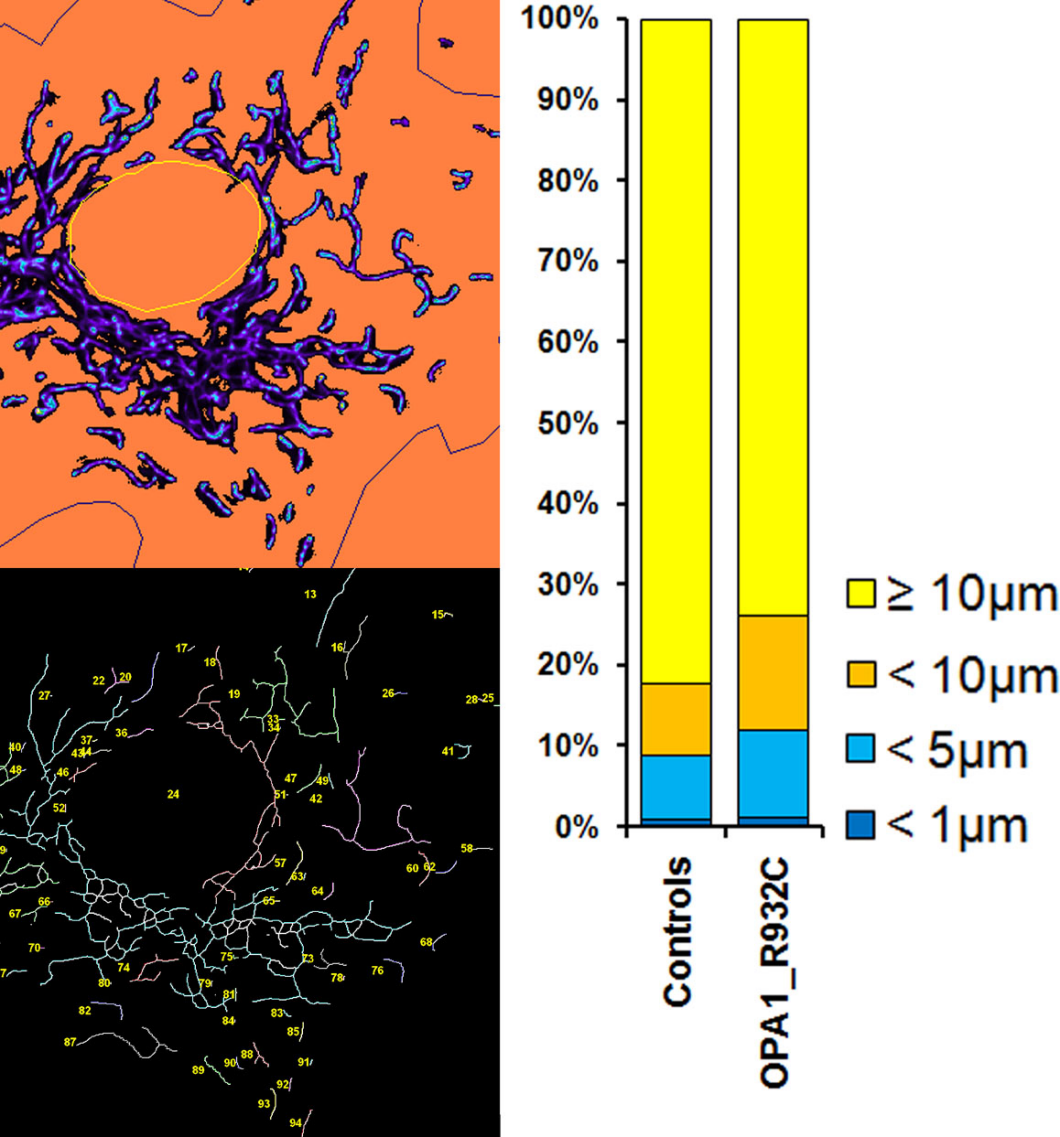
Quantification of mitochondrial shape showed that the patient’s fibroblasts (OPA1_R932C) contained a higher proportion of short mitochondria than controls (Appendix 3).

The quantification of mitochondria length distribution showed a larger proportion of short mitochondria in the patient’s fibroblasts than in controls (Figure 6), as usually observed in fibroblasts from patient with OPA1 mutations. However, this observation is not correlated to a fragmented network, which could be shown in apoptotic cells. Culture cell studies performed on patient’s cells did not show a higher rate of apoptosis nor a greater number of necrotic cells as compared to controls. These results were obtained using a viability test (Trypan blue exclusion test, data not shown).

**Figure 7:**
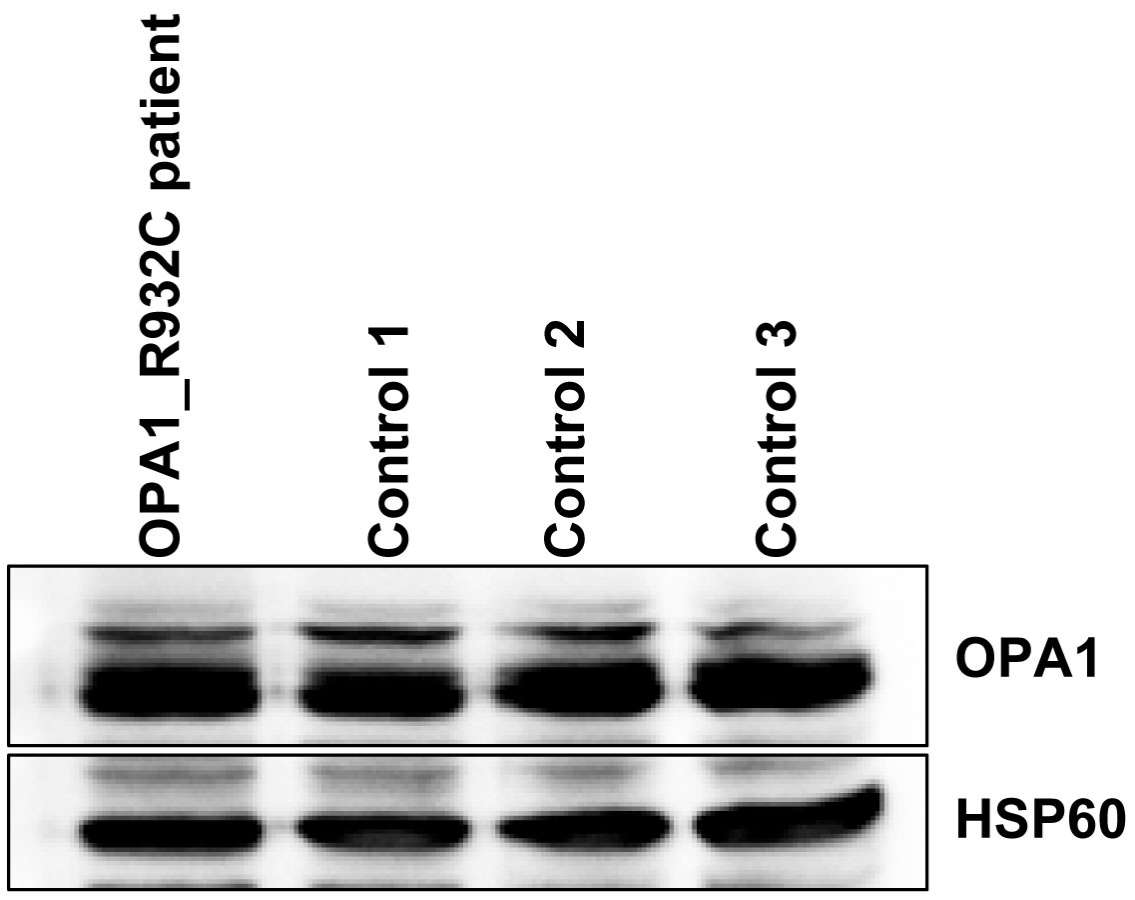
Expression of OPA1 protein in fibroblasts from the OPA1_R932C patient and from controls. Cellular extracts were analyzed by western blotting using antibodies against OPA1 and HSP60 as described in materials and methods. HSP60 was used as a mitochondrial marker and as a control for protein loading.

In spite of the mutation, there was no quantitative modification of OPA1 expression in the patient’s fibroblasts. We also observed no alteration of the OPA1 expression pattern in these cells (Figure 7).

## Discussion

ADOA is an inherited optic neuropathy that leads to reduced visual acuity. It is classically characterized by the insidious onset of visual impairment in early childhood with moderate to severe loss of visual acuity, temporal optic disc pallor, color vision deficits, and centrocecal scotoma of variable density [28]. Histopathologic studies have shown that the fundamental underlying defect is degeneration of the retinal ganglion cells, followed by ascending optic atrophy [1]. In about 70% of the cases, ADOA is due to mutations in the nuclear *OPA1* gene situated on chromosome 3q28-q29 [7,8,29]. The OPA1 protein is a dynamin-related GTPase targeted to mitochondria, where it is anchored to the mitochondrial inner membrane [11,30]. Our analysis of the mitochondrial network showed that fibroblasts with the c.2794C>T mutation contained a larger proportion of short mitochondria than controls (Figure 6). This finding is in accordance with the functional involvement of OPA1 in the structure of the mitochondrial network and with the alterations of mitochondrial structure observed in fibroblasts carrying OPA1 mutations [23].

Since the first description of the disease [6], the classic presentation of ADOA has widened to include less typical features. Indeed, gender-related variations and intrafamilial phenotypes heterogeneity of ADOA have been reported [31]. Visual loss has been found to be more severe in affected males than in females [31]. The expression of the disease is highly variable, even within a given family, and asymptomatic carriers can be found in some families [5,32]. The availability of molecular testing has revealed phenotypic variations, such as severe congenital forms of optic atrophy [16], ADOA associated with sensorineural deafness [18], and ADOA associated with more complex, severe multi-organ phenotypes [14,15]. However, no correlation has been found between the degree of visual impairment and the location or type of mutation in *OPA1* [33,34]. The cause of this variable phenotypic expression remains to be elucidated but could be due to various genetic or environmental factors. Other genes encoding proteins involved in the modulation of mitochondrial bioenergetics and structure may influence the phenotypic expression of the disease. For instance it has been shown that the mitochondrial DNA, which encodes respiratory chain subunits, can influence the expression of ADOA [35]. Environmental factors such as drugs with mitochondrial toxicity could also be involved in the penetrance and expressivity of the disease.

Our case report demonstrates the existence of a new mode of onset of ADOA related to OPA1 mutation with an acute and a late onset that contrast with the earlier and insidious onset of the disease classically observed and described. The acute-onset presentation of optic atrophies is usually associated with Leber hereditary optic neuropathy, which remains the main differential diagnosis of ADOA. This mitochondrial DNA mutation-related disease is classically associated with acute or subacute bilateral optic atrophy occurring in the third decade of life, and preferentially affecting males [36]. Our observation shows that acute-onset presentations should benefit from *OPA1* analysis when LHON mutations have been excluded. Late-onset cases of optic atrophies are rarely considered to be caused by primary hereditary diseases but frequently believed to be due to secondary causes. Our case shows that *OPA1* analysis can be useful in late-onset cases of ADOA when the main secondary causes of optic atrophies have been excluded.

The novel OPA1 mutation is associated with a mitochondrial energy defect involving reduced ATP synthesis and a cytochrome c oxidase deficiency. A similar, but less marked, biochemical phenotype has been previously described in fibroblasts from patients with ADOA carrying OPA1 mutations; in this study the reduced efficiency of oxidative phosphorylation was found to be compensated by a higher rate of mitochondrial respiration to maintain ATP synthesis [22]. In our case, the marked decrease in COX activity might limit the possibility of compensation by a higher rate of mitochondrial respiration.

In conclusion, we report a novel OPA1 mutation in a patient with ADOA characterized by sporadic, acute, and late-onset presentation. This finding, taken together with several recently published observations, demonstrates that the clinical spectrum of OPA1-associated optic neuropathies is much larger than previously believed. The systematic search for *OPA1* mutations should therefore prove useful in unexplained and atypical cases of optic atrophy.

## Appendix 1. Amplification and sequencing primers for OPA1 gene.

The nucleotide sequence of the primers used to amplify and sequence the 30 OPA1 coding exons, including exon-intron junctions, is given according to the GenBank reference sequence NC_000003.10. To access the data, click or select the words “Appendix 1.” This will initiate the download of a pdf file that contains the information.

## Appendix 2. Amplification and sequencing primers for mtDNA.

The nucleotide sequence of the primers used to amplify and sequence the entire mitochondrial DNA is given according the Revised Cambridge Reference Sequence of the Human Mitochondrial DNA (RefSeq: AC_000021.2) numbering, described as being the “Light strand.” Primers in Italic were those used for DNA amplification. To access the data, click or select the words “Appendix 2.” This will initiate the download of a pdf file that contains the information.

## Appendix 3. Representative deconvolved images used to quantify mitochondrial length:

Mitochondrial length was determined using integrated morphometric analysis of the Mitotracker®-labeled regions created after deconvolution. The Figure shows examples of the mitochondrial network observed in the patient's fibroblasts. To access the data, click or select the words “Appendix 3.” This will initiate the download of a pdf file that contains the information.
